# Institutional Questions

**Published:** 1899-10-14

**Authors:** 


### INSTITUTIONAL QUESTIONS.

### Ambulances.

M. O. H. writes: Will you kindly tell me where the street ambulances in use in London can be obtained, and if one of these would be as good as any for emergency service at a small country hospital?

You mean, no doubt, the "Hospitals' Association" litter generally in use for the street ambulance service in London. It is made by Messrs. Carter, 6a, New Cavendish Street, London, W., and would probably well serve your purpose. Write to Messrs. Carter for catalogue and full particulars.

### District Work and Sanitary Law.

"Sanitas" writes: Can you advise in the following circumstances? A fully-qualified nurse of many years' experience contracts diphtheria while nursing through an epidemic in her district, caused by the terribly unhealthy condition of the village. The drainage and water supply are most unsatisfactory, and the death-rate very high. The doctor is the medical officer of health. The nurse works under a committee, but no one seems to recognise the fatal consequences of the general neglect of sanitation. Could application be made to any public sanitary body, who would take the matter up and send an inspector?

The proper course in such circumstances as you describe, whether in town or country, would seem to be to call the attention of the sanitary inspector to the sanitary defects, and, if necessary, to apply to the medical officer. It is the duty of the sanitary authority (probably in your case the District Council) to compel those responsible—landlord or occupier, as the case may be—to abate any nuisance which is proved to exist. Failing the sanitary authority, complaint might be made to the County Council or the magistrates, or a petition be sent to the Local Government Board; the latter body will, under certain circumstances, send an inspector to conduct an inquiry regarding the sanitary condition of a village or district. There is no doubt considerable backwardness amorg some sanitary authorities in putting the law respecting nuisances into operation, but if, when complaint is made, it is at the same time made clear that things will be carried further if no notice be taken, the result is generally satisfactory. The Local Government Board, acting through its medical department, is to be looked upon as the final appeal in sanitary matters. To take action on her own account in this matter might be somewhat outside the duty of a nurse working under a committee, but it would be undoubtedly open to her to demonstrate emphatically to the members of her committee, and generally to the people in the village, that it is absolutely their own fault if the continuance of any nuisance be allowed without protest. The real bar to the effectual application of the sanitary law is the apathy of the inhabitants of any district or neighbourhood. If two or three residents will set to work with energy they can speedily set the whole machinery in motion, thereby doing much good, and usually earning much enmity.

